# Lipids as paleomarkers to constrain the marine nitrogen cycle

**DOI:** 10.1111/1462-2920.13682

**Published:** 2017-02-28

**Authors:** Darci Rush, Jaap S. Sinninghe Damsté

**Affiliations:** ^1^ Department of Marine Microbiology and Biogeochemistry, NIOZ Royal Netherlands Institute for Sea Research and Utrecht University Den Burg P.O. Box 59 1790 AB The Netherlands; ^2^ School of Civil Engineering and Geosciences Newcastle University Newcastle‐upon‐Tyne NE1 7RU United Kingdom; ^3^ Department of Earth Sciences, Faculty of Geosciences Utrecht University TA Utrecht P.O. Box 80.121, 3508 The Netherlands

## Abstract

Global climate is, in part, regulated by the effect of microbial processes on biogeochemical cycling. The nitrogen cycle, in particular, is driven by microorganisms responsible for the fixation and loss of nitrogen, and the reduction‐oxidation transformations of bio‐available nitrogen. Within marine systems, nitrogen availability is often the limiting factor in the growth of autotrophic organisms, intrinsically linking the nitrogen and carbon cycles. In order to elucidate the state of these cycles in the past, and help envisage present and future variability, it is essential to understand the specific microbial processes responsible for transforming bio‐available nitrogen species. As most microorganisms are soft‐bodied and seldom leave behind physical fossils in the sedimentary record, recalcitrant lipid biomarkers are used to unravel microbial processes in the geological past. This review emphasises the recent advances in marine nitrogen cycle lipid biomarkers, underlines the missing links still needed to fully elucidate past shifts in this biogeochemically‐important cycle, and provides examples of biomarker applications in the geological past.

## Biomarker lipids and the marine nitrogen cycle

**Figure 1:**
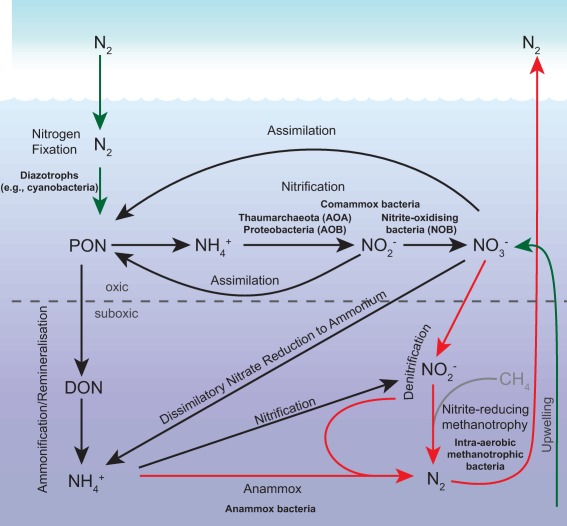
The marine nitrogen cycle. Microorganisms with known biomarkers are indicated next to their transformation pathways. Losses of nitrogen are indicated by red arrows, sources in green. N_2_: dinitrogen gas; PON: particulate organic nitrogen; DON: dissolved organic nitrogen; ${\text{NH}}_{4}^{+}$: ammonium; ${\text{NO}}_{2}^{-}$: nitrite; ${\text{NO}}_{3}^{-}$: nitrate. Figure adapted from Arrigo, 2005.

Nitrogen is vital to the building blocks of life (e.g. nitrogen is a key element in amino acids, nucleic acids, and membrane lipids). The largest marine pool of nitrogen is dissolved dinitrogen (N_2_). However, few organisms in the ocean are capable of utilising N_2_; non‐diazotrophic microbes must depend on the less substantial pools of bio‐available nitrogen [i.e. nitrate ( ${\text{NO}}_{3}^{-}$); nitrite ( ${\text{NO}}_{2}^{-}$); ammonium ( ${\text{NH}}_{4}^{+}$)] to sustain their activity (Fig. 1). The concentration of these different forms of nitrogen varies spatially and temporally in the ocean. In general, the highest concentrations of ${\text{NO}}_{2}^{-}$ and ${\text{NH}}_{4}^{+}$ are found in the surface waters where they are assimilated by phytoplankton to form a pool of organic nitrogen. The concentrations of these two species decrease significantly at the base of the euphotic zone, as they are converted into ${\text{NO}}_{3}^{-}$ by nitrifying microorganisms. ${\text{NO}}_{3}^{-}$ on the other hand accumulates with water depth and is usually present an order of magnitude higher than ${\text{NO}}_{2}^{-}$ and ${\text{NH}}_{4}^{+}$ (Gruber, 2008). The fate of nitrogen in deeper waters can either be towards burial as particulate matter, or complete loss as it is converted back to N_2_ in anoxic conditions. Nevertheless, it must be emphasised that the presence of these bio‐available forms of nitrogen in the ocean is inconsistent, and their concentrations in the water column are habitually low.

Nitrogen is often the limiting nutrient for primary productivity in marine environments, and our understanding of the marine carbon pump inherently depends on what we know of the nitrogen cycle. Since the global inception of the Haber‐Bosch process in the early 20th century, anthropogenic activity has significantly influenced the marine nitrogen cycle. Run‐off from fertilisers in coastal areas causes eutrophication of the water column, leading to oceanic dead zones (Altieri and Gedan, 2015), and changes in the extent and vertical structure of oxygen minimum zones (OMZs) (Paulmier and Ruiz‐Pino, 2009; Wright *et al*., 2012). Besides the obvious repercussions on ecology and climate, anthropogenic nitrogen input also greatly affects local economies by decreasing the available stock of commercial fish (Lavin *et al*., 2006; Rabotyagov *et al*., 2014).

The transformations between chemical species of nitrogen are primarily regulated by microbial activity, with specialised organisms performing each process (Fig. 1). Nitrogen cycle microorganisms are often taxonomically divided, and many have structurally unique lipids, known as biomarkers (Peters *et al*., 2005). By employing biomarker lipids, we can determine the contributions of different nitrogen cycle processes through Earth's history. Biomarkers are especially useful in reconstructing paleo‐environments where genetic information has been degraded (cf. Hofreiter *et al*., 2001). Subsequently, this biomarker information can be used to better predict modern and future nitrogen and carbon cycle variations by constraining biogeochemical models of the past [e.g. during the Cenomanian‐Turonian Ocean Anoxic Event (OAE 2); Monteiro *et al*., 2012].

Biomarkers exist for many of the key nitrogen biogeochemical processes. However, there are some glaring gaps, notably on the anoxic side of the nitrogen cycle. OMZs are found in the marine water column underlying productive photic zones, and are hotbeds for anaerobic nitrogen cycling processes, most of which result in net marine nitrogen loss through nitrogenous gas production. It has been estimated that half of the nitrogen loss in the ocean occurs within these OMZs (Codispoti *et al*., 2001; Lam *et al*., 2009). In our current dynamic ocean climate of increasing temperature and decreasing ventilation, OMZs are expanding (Wright *et al*., 2012). It is imperative that we be able to predict how past perturbations in ocean biogeochemistry, especially within OMZs, affected the sensitivity of the system.

Several review papers have highlighted the marine nitrogen cycle (Gruber, 2004, 2008; Arrigo, 2005; Capone, 2008; Lam and Kuypers, 2011; Zehr and Kudela, 2011; Voss *et al*., 2013; Ward and Jensen, 2014), but none have focused on nitrogen cycle biomarkers. Here, we describe the biomarker lipids known for key processes in the marine nitrogen cycle, how they can be applied to cycling in the geological past, and draw attention to the processes within the nitrogen cycle that still need resolved biomarkers.

## Bringing nitrogen in: Diazotrophic organisms in the marine system

N_2_ accounts for 94% (10^7^ Tg N) of the nitrogen in the ocean (Gruber, 2008). Diazotrophy, or nitrogen‐fixation, is essential to the fertilisation of ocean surface waters in oligotrophic regions, where there are no other significant inputs of nitrogen (Capone, 2001; Mahaffey *et al*., 2005). N_2_‐fixation has been estimated to contribute 36–50% of the nitrogen used by microbes in the Pacific and Atlantic gyres (Carpenter *et al*., 1999; Dore *et al*., 2002). The most important marine diazotrophs belong to the phylum Cyanobacteria (Carpenter and Romans, 1991; Capone *et al*., 1997; Karl *et al*., 1997; Gallon, 2001), and by far the most well‐studied species are the filamentous *Trichodesmium* spp. However, unicellular cyanobacteria (Martínez‐Pérez *et al*., 2016) and non‐cyanobacteria diazotrophs (Farnelid *et al*., 2011) may also play an important role in N_2_‐fixation in marine systems. Diazotrophic organisms use the enzyme nitrogenase to break the triple bonds between the N atoms of N_2_. Nitrogenase is inactive in the presence of oxygen, a fact that is problematic to the surface dwelling, primarily photoautotrophic, microorganisms using nitrogenase. Diazotrophs compensate for this in different ways. Some use the light/dark cycle to spatially separate these processes. Others use physical separation by compartmentalising diazotrophy into specialised cells. Below, we discuss two groups of diazotrophy biomarkers.

### Bacteriohopanepolyols of cyanobacteria

**Figure 2:**
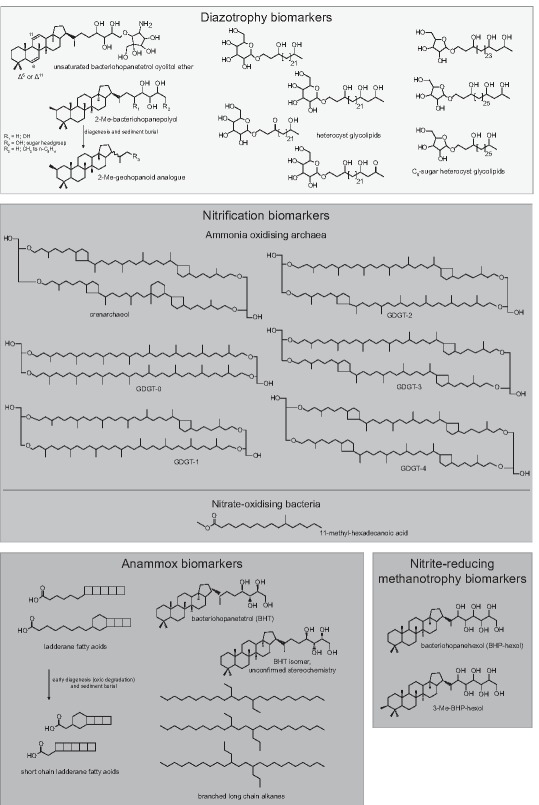
Biomarkers of microbial processes in the marine nitrogen cycle. Diazotrophy biomarkers: unsaturated bacteriohopanetetrol cyclitol ether; 2‐methyl‐bacteriohopanepoyols are diagenetically altered in sediment burial to 2‐methyl‐geohopanoids; heterocyst glycolipids with C_6_ and C_5_ sugar head groups. Nitrification biomarkers: crenarchaeol and GDGT‐0‐4; 11‐methyl‐hexadecanoic acid. Anammox biomarkers: ladderane fatty acids undergo oxic degradation into short chain ladderane fatty acids; bacteriohopanetetrol stereoisomer; anammox bacterial biomass is thermally altered to produce branched long chain alkanes. Nitrite‐reducing methanotrophy biomarkers: bacteriohopanehexol; 3‐methyl‐bacteriohopanehexol.

Cyanobacteria are considered to be the most environmentally significant source of bacteriohopanepolyol lipids (BHPs) (Farrimond *et al*., 1998; Talbot and Farrimond, 2007). BHPs are the precursors to the most ubiquitous lipids found in geological materials: geohopanoids (e.g. Ourisson and Albrecht, 1992). BHPs are pentacyclic C_30_ triterpenoids linked to a polyfunctionalised side chain with e.g. hydroxyl, amine, or cyclic groups (Fig. 2). Although not all cyanobacteria synthesise BHPs, and many synthesise non‐source specific BHPs, [e.g. bacteriohopanetetrol (BHT) and aminobacteriohopanetriol (aminotriol)], some marine N_2_‐fixing species of cyanobacteria have been shown to synthesise more characteristic BHPs. C‐2 methylated BHPs (Fig. 2) were found in *Trichodesmium* and *Crocosphaera*, both ubiquitous marine species (Talbot *et al*., 2008). C‐2 methylated hopanes (the diagenetic product of BHPs) were originally thought to be a biomarker for cyanobacteria, and have been used previously to indicate the rise of oxygenic photosynthesis (Summons *et al*., 1999) as well as the importance of N_2_‐fixation at times of stratified oceans (e.g. Cretaceous black shales; Kuypers *et al*., 2004). However, other bacteria have also been found to synthesise C‐2 methylated BHPs [i.e. anoxygenic phototroph *Rhodopseudomonas palustris* TIE‐1 (Rashby *et al*., 2007), *Methylobacterium* spp. (Bisseret *et al*., 1985; Knani *et al*., 1994) and *Bradyrhizobium* spp. (Bravo *et al*., 2001)]. Furthermore, the gene coding for the enzyme responsible for C‐2 methylation in BHPs was found to occur across the bacterial domain (Welander *et al*., 2010). Thus, using the C‐2 methylation in hopanoids as a biomarker for cyanobacteria input and the onset of atmospheric oxygen is complicated.

A more appropriate BHP biomarker for marine N_2_‐fixation may be BHT cyclitol ether (BHT‐CE; Fig. 2) with an unsaturation at the C‐6 or C‐11 position. Unsaturated BHT‐CE is synthesised by *Trichodesmium* sp. (Talbot *et al*., 2008), one of the most prolific and biogeochemically‐important marine cyanobacteria. However, this lipid has also been found in acetic acid bacterial cultures (Simonin *et al*., 1994; Herrmann *et al*., 1996; Talbot *et al*., 2007), and several strains of *Burkholderia cepacia* isolated from acetic grasslands (Cvejic *et al*., 2000). Thus, care must be taken when applying unsaturated BHT‐CE as a biomarker for *Trichodesmium*. As it is unlikely that either of these other types of bacteria proliferate in open marine settings, unsaturated BHT‐CE was successfully applied as a biomarker for *Trichodesmium* in sediments from the Congo deep sea‐fan to interpret the past marine nitrogen cycle (Handley *et al*., 2010). Unsaturated BHT‐CE was present in unprecedented abundance at a specific sedimentary horizon (480–610 ka). Here, the abundance of unsaturated BHT‐CE was interpreted to be caused by a decrease in nutrient supply, which resulted in increased N_2_‐fixation by diazotrophic cyanobacteria.

### Heterocyst glycolipids

A large group of diazotrophic cyanobacteria contain heterocysts, specialised cells where diazotrophy occurs under induced low oxygen conditions. To maintain anoxia, heterocystous cyanobacteria surround these cells with a thick enveloppe limiting the diffusion of gases (Walsby, 1985; Murry and Wolk, 1989), which is made up of glycolipids known as heterocyst glycolipids (Nichols and Wood, 1968; Bryce *et al*., 1972; Lambein and Wolk, 1973). Heterocyst glycolipids consist of sugar functionalities glycosidically bound to long‐chain (C_26_–C_32_) diols, triols, keto‐ols, or keto‐diols (Fig. 2). The distribution of heterocyst glycolipids in cyanobacteria was screened using high‐performance liquid chromatography coupled to electrospray ionisation tandem mass spectrometry, and was found to vary according to taxonomic groups (Bauersachs *et al*., 2009a, 2009b). For example, species belonging to the family *Nostocaceae* synthesise the glycolipids 1‐(O‐hexose)−3,25‐hexacosanediol and 1‐(O‐hexose)−3‐keto‐25‐hexacosanol, whereas members of the *Calothrix* genus primarily synthesise 1‐(O‐hexose)−3,25,27‐octacosanetriol and 1‐(O‐hexose)−3‐keto‐25,27‐octacosanediol.

In marine systems, heterocystous cyanobacteria are also commonly found in symbiosis with diatoms (Villareal, 1992). Schouten *et al*. (2013a) found that these endosymbiotic marine cyanobacteria species synthesise heterocyst glycolipids with distinct C‐5 sugar moieties (Fig. 2). These have been found in suspended particulate matter and surface sediments from the marine Amazon plume region, and proposed to be specific biomarkers for marine endosymbiotic heterocystous cyanobacteria (Bale *et al*., 2015).

## Converting waste into nutrients: Nitrification

Most fixed organic nitrogen is remineralised back into ${\text{NH}}_{4}^{+}$ by heterotrophic bacteria. This pool of reduced nitrogen is then oxidised by nitrifying microorganisms (Fig. 1). Nitrification is the primary source of oxidised nitrogen species in the ocean, converting reduced ${\text{NH}}_{4}^{+}$ to oxidised ${\text{NO}}_{2}^{-}$ and ${\text{NO}}_{3}^{-}$. Traditionally, nitrification is divided into two distinct steps, performed by different groups of microorganisms: (1) ammonia oxidation to nitrite [initially thought to be performed by ammonia‐oxidising members of Proteobacteria (AOB), and since expanded to include ammonia‐oxidising archaea (AOA)], and (2) nitrite oxidation to nitrate (nitrite‐oxidising bacteria; NOB) (Ward, 2013a). Recently, specific species of the bacterial genus *Nitrospira* have been identified performing both of the nitrification steps completely [complete ammonia oxidation (comammox); Daims *et al*., 2015; van Kessel *et al*., 2015; Pinto *et al*., 2016].

### Lipids of ammonium‐oxidising bacteria (AOB)

The most common AOBs in natural environments belong to the genus *Nitrosomonas*. However, the lipids of *Nitrosomonas* spp. are undiagnostic, including generic BHPs (aminobacteriohopanetriol, diploptene) and fatty acids (unsaturated‐, *cis‐*9‐ and *trans*−9‐hexadecanoic acids) (Sakata *et al*., 2008).

### Glycerol dibiphytanyl glycerol tetraethers (GDGTs) of AOA

The discovery that archaea, which dominate the marine mesopelagic zone (Karner *et al*., 2001), were responsible for a significant amount of ammonia oxidation in the ocean was revolutionary (Francis *et al*., 2005; Könneke *et al*., 2005; Wuchter *et al*., 2006). To date, the phylum Thaumarchaeota contains three orders of autotrophic ammonia oxidisers, all of which synthesise a suite of archaeal membrane lipids known as glycerol dibiphytanyl glycerol tetraethers (GDGTs). The stereochemical structure of crenarchaeol, the one abundant GDGT found so far exclusively in Thaumarchaeota was first described by Sinninghe Damsté *et al*. (2002a). Crenarchaeol was named for the ‘Creanarchaeota’ phylum, the mesopholic branch of which has since been reclassified as ‘Thaumarchaeota’ (Brochier‐Armanet *et al*., 2008; Spang *et al*., 2010). Crenarchaeol contains two cyclopentyl moieties in one of its isoprenoid chains, and one cyclohexyl moiety and two cyclopentyl moieties in its other chain (Fig. 2). AOA also synthesise common and thus non‐source‐specific GDGT‐0 and GDGT‐1‐4 (Fig. 2). The application of crenarchaeol as a biomarker for AOA has been challenged by the suggestion that Marine Group II Euryarchaeota may synthesise this lipid (Lincoln *et al*., 2014). However, there are no cultivated relatives of Marine Group II. Currently, both phylogenomic analysis of genes responsible for archaeal lipid biosynthesis (Villanueva *et al*., 2017) and environmental data (cf. Schouten *et al*., 2014) point to Thaumarchaeota being the sole source of crenarchaeol. It should be noted that some Thaumarchaeota are not obligate chemolithoautotrophs (Mußmann *et al*., 2011), and the presence of thaumarchaeal GDGTs does not necessarily mean ammonia oxidation has been occurring in the environment.

Arguably the most important application of AOA lipids is to paleo‐thermometry. The number of rings in the GDGT structures is temperature‐dependent, a fact that has been exploited to create the TEX_86_ paleo‐thermometer (Schouten *et al*., 2002; Kim *et al*., 2010). The TEX_86_ proxy has been applied extensively to determine sea surface temperatures (SST) of paleo‐environments up until the Middle Jurassic (160 Ma) (Jenkyns *et al*., 2012; for full review, see Schouten *et al*., 2013b). Some research has contested whether TEX_86_ does in fact reflect SST (Shah *et al*., 2008; Ho and Laepple, 2016). The paradox of TEX_86_ is that though it seemingly records surface temperature, the Thaumarchaeota responsible for synthesising GDGTs reside in both the shallow and deep water column. Ammonia monoxygenase (amoA) gene sequences have established the division into ‘shallow’ and ‘deep’ water clusters of AOA Thaumarchaota (e.g. Francis *et al*., 2005; Hallam *et al*., 2006; Hu *et al*., 2011). This trend was also observed recently in the production of GDGTs within the water column by evaluating a key gene involved in the lipid biosynthetic pathway coding for GDGT precursor geranylgeranylglyceryl phosphate (GGGP) synthase in Thaumarcheaota (Villanueva *et al*., 2015). These authors observed a positive correlation between an increase of GGGP synthase and the relative proportion of GDGT‐2 with water depth, suggesting a niche differentiation in GDGT distributions. Interestingly, this depth speciation was not observed in 16S gene‐based phylogeny (Schouten *et al*., 2012). Ammonia availability was suggested to be responsible for the niche partitioning of thaumarchaeota in the Arabian Sea (Villanueva *et al*., 2015). The ‘deep’ cluster of AOA on the Portuguese continental margin was found to contribute significantly to the GDGTs present in sediments at greater water depth, implying this non‐surface population does have an influence on TEX_86_ (Kim *et al*., 2016). Hurley *et al*. (2016) recently suggested that the distribution of GDGTs of thaumarchaeota may be dependant on the nitrification rate. These studies merely highlight the further work required to understand the signal that TEX_86_ records.

### Fatty acids of nitrite‐oxidising bacteria (NOB)

*Nitrospina* spp. are the most abundant NOB in marine systems, and dominate microaerophilic zones of OMZs (Lücker *et al*., 2013). Fatty acids of four genera of NOB (*Nitrobacter*, *Nitrococcus*, *Nitrospina*, and *Nitrospira*) have been described, and each genus appears to have its own distinct profile (Auran and Schmidt, 1972, 1976; Lipski *et al*., 2001) (Fig. 2). Although fatty acids are typically not specific enough to be applied as biomarkers, these differences would enable differentiation between NOB bacteria in particular environments. A novel fatty acid, 11‐methyl‐hexadecanoic acid, was identified in *Nitrospira moscoviensis* (Lipski *et al*., 2001). Unfortunately, however, fatty acids are not recalcitrant enough to endure diagenesis in buried marine sediments, and hence, fatty acids can typically not be used in sediments older than ca. 1 Ma.

Given that the comammox bacteria described performing complete nitrification belong to the genus *Nitrospira*, it is possible that comammox species synthesise 11‐methyl‐hexadecanoic acid. However, non‐comammox species of *Nitrospira* also synthesise 11‐methyl‐hexadecanoic acid (Lebedeva *et al*., 2011), showing that this fatty acid is not an appropriate biomarker for comammox. The *Nitrospira* genus is diverse. *Nitrospira* has at least six sub‐lineages (Daims *et al*., 2001), and has been found in various natural environments (e.g. marine, Watson *et al*., 1986; fresh water, Daims *et al*., 2001; and terrestrial, Lebedeva *et al*., 2011; Pester *et al*., 2014). Lipski *et al*. (2001) found that the two species they studied belonging to *Nitrospira* already showed variations between their lipids. Thus, it is possible that comammox *Nitrospira* spp. have their own distinct fatty acid lipid profile, which has yet to be characterized.

## Closing the circle: Denitrifying processes return nitrogen to the atmosphere

In anoxic environments, the system shifts towards favouring organisms that use reaction pathways resulting in net nitrogen loss through the production of N‐containing gases, i.e. nitrous oxide (N_2_O) and N_2_ (Mulder *et al*., 1995; Zumft, 1997). There are two major pathways of nitrogen loss from the marine environment: denitrification and anaerobic ammonium oxidation (anammox) (Fig. 1). In modern marine environments, the production of N_2_ by anammox and denitrification represents an important sink of bio‐available nitrogen.

Before the discovery of anammox in a waste water treatment plant (Mulder *et al*., 1995), ${\text{NH}}_{4}^{+}$ was thought to be unreactive in anoxic systems. However, in the following decades the bacteria that anaerobically oxidise ${\text{NH}}_{4}^{+}$ using ${\text{NO}}_{2}^{-}$ have been found to be prevalent in marine systems (Dalsgaard and Thamdrup, 2002; Kuypers *et al*., 2003; Capone, 2008; Gruber, 2008), especially within OMZs (Kuypers *et al*., 2005; Hamersley *et al*., 2007; Lam *et al*., 2009; Sollai *et al*., 2015). Anammox bacteria are slow‐growing, taking weeks to divide (Strous *et al*., 1999), which might be to their advantage in certain systems as they may not have to respond to sudden changes in substrate concentration. For example, the anammox reaction was found consistently, though at low rates, within the Eastern Tropical South Pacific OMZ, whereas the presence of denitrification was sporadic (Dalsgaard *et al*., 2012). This could indicate that anammox represents a constant background loss of nitrogen from anoxic systems.

### Ladderane and BHT lipids of anammox bacteria

One of the most challenging puzzles in membrane lipid composition of the last 15 years was the stereochemical structure of lipids found exclusively in anammox bacteria. Strained cyclobutyl moieties require such a high amount of energy to synthesise that they were not thought to exist in nature. However, isolated lipids from anammox bacterial enrichments proved to have three or five concatenated cyclobutyl moieties (Fig. 2; Sinninghe Damsté *et al*., 2002b), and were named ‘ladderane lipids’ for their 3‐dimensional structure, which was later confirmed by total synthesis (Mascitti and Corey, 2004; Mercer *et al*., 2016). Ladderanes play an important role within the anammox cells. These lipids create a dense membrane bilayer that prevents the diffusion of the toxic intermediate gas (hydrazine) of the anammox reaction into other cell functions. The anammox reaction is thus entirely contained within the organelle‐like anammoxosome. Originally thought to be specific to the anammoxosome, ladderane lipids have subsequently been found in other anammox cell fractions (Neumann *et al*., 2014). These results seem to indicate that the anammox bacteria do not possess a mechanism to sort their lipids. Nevertheless, ladderanes are excellent biomarkers because they are uniquely synthesised by anammox bacteria.

Unfortunately, both the strain on the cyclobutyl moieties in addition to the carboxyl functionality of ladderane fatty acids means that these biomarkers do not tolerate diagenesis to a significant extent, and have not been found in mature marine sediments. Recently, a relatively rare stereoisomer of BHT (BHT isomer) was found to be synthesised in high abundance by marine anammox bacteria (Fig. 2; Rush *et al*., 2014a). This BHP was also found in sediments from an anoxic marine fjord. Considering BHPs have been found in sediments of 56 Ma old (Talbot *et al*., 2016), BHT isomer shows potential to fill the gap as a biomarker for anammox in sediments that are not yet thermally mature, but which are older than the limit of diagenetic alteration for ladderanes.

### Nitrite‐reducing methanotrophy biomarkers

*Methylomirabilis oxyfera* is an exceptional methanotroph that produces its own oxygen from ${\text{NO}}_{2}^{-}$ via the production of NO by nitrite reductase (Ettwig *et al*., 2010). This O_2_ is subsequently used to oxidise methane. *M. oxyfera* was shown to synthesise characteristic BHP biomarkers: bacteriohopanehexol (BHP‐hexol) and 3‐Me‐BHP‐hexol (Fig. 2; Kool *et al*., 2014). Although the significance of this process within the marine nitrogen cycle is currently unknown, intra‐aerobic methanotrophy has recently been detected in marine OMZs, suggesting it may play a role in the nitrogen cycle of these environments (Padilla *et al*., 2016). Using 3‐Me‐BHP‐hexol, we may be able to understand past ${\text{NO}}_{2}^{-}$ availability in dynamic OMZ systems, where up to 50% of marine bioavailable nitrogen loss occurs (Codispoti *et al*., 2001; Lam *et al*., 2009).

### Anaerobic nitrogen cycle processes without specific biomarker lipids

The importance of dissimilatory nitrate reduction to ammonium (DNRA; Fig. 1) in marine systems is unresolved. DNRA was proposed to be an important source of ${\text{NH}}_{4}^{+}$ in the Peruvian OMZ (Lam *et al*., 2009), but was not detected within the Chilean OMZ (De Brabandere *et al*., 2014).There are currently no biomarker lipids available to trace past shifts of DNRA.

Perhaps the most versatile anaerobic nitrogen cycle process is denitrification, where ${\text{NO}}_{3}^{-}$ is reduced via ${\text{NO}}_{2}^{-}$, NO, and N_2_O to N_2_ (Fig. 1). However, this process is not related to a specific taxonomic group (e.g. autotrophic and heterotrophic bacteria, archaea, and foraminifera can all perform denitrification; Zumft, 1997; Risgaard‐Petersen *et al*., 2006), which severely complicates finding biomarkers that are specific to denitrification. It is thus more likely that if we are going to trace denitrification using lipids, it will be with a suite of biomarkers related to individual denitrifiers. Another possible avenue is using anammox biomarkers to infer denitrification. Ward (2013b) proposed that the proportion of anammox is thermodynamically linked to denitrification, where the latter accounts for 70% of N loss from the ocean. If this proves to hold true over nitrogen cycle history, we could reconstruct total nitrogen loss due to anammox and denitrification solely using anammox biomarkers.

## Application of biomarkers to interpret nitrogen cycling in the geological past

Nitrogen cycle biomarkers can be applied to the modern ocean, complementing molecular, genetic, and isotope‐labeling approaches (e.g. Kuypers *et al*., 2003, 2005; Hamersley *et al*., 2007; Pitcher *et al*., 2011). However, such biomarkers are especially beneficial for the geological past, where these other techniques can no longer be used. Below, we discuss four examples of nitrogen cycle biomarker application.

## Ladderane lipids: The importance of anammox in the past arabian sea OMZ

**Figure 3:**
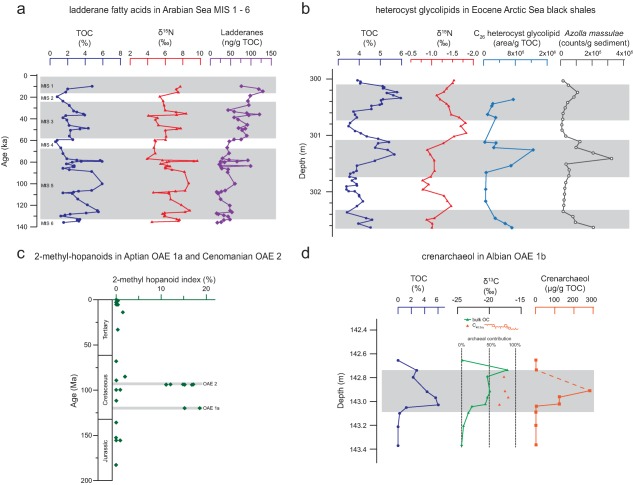
Biomarker evidence for paleo‐nitrogen cycling processes from (a) ladderane fatty acid abundances (Jaeschke *et al*., 2009) of Arabian Sea sediments underlying the oxygen minimum zone (core MD04‐2879) over the past 140 ka, spanning Marine Isotope Stages (MIS) 1 to 6; (b) heterocyst glycolipid abundances (Bauersachs *et al*., 2010) and *Azolla massulae* counts (Speelman *et al*., 2009) of Eocene Arctic Ocean sediments including black shale intervals (Integrated Ocean Drilling Project Expedition 302, ACEX); (c) 2‐methyl‐hopanoids indices (Kuypers *et al*., 2004) of organic‐rich sediment from the last 200 Ma, including the Cenomanian ocean anoxic event (OAE 2) (Deep Sea Drilling Project Sites 144, 367, and 603B, proto North Atlantic) and Aptian OAE 1a (Cismon apticore, Southern Alps, Italy, Aptian Thetys; Deep Sea Drilling Project Site 463, Pacific Ocean). Values represent averages of 1‐24 samples.; (d) crenarchaeol abundances and carbon isotopic values of tricyclic biphytanes (C_40:3cy_) derived from archaea (Kuypers *et al*., 2001) of Albian OAE 1b (Ocean Drilling Project site 1049C). Warm MIS and anoxic events are indicated as grey shaded areas in the graphs. TOC: total organic carbon; *δ* ^15^N: bulk sediment nitrogen isotope value (in per mil vs. N_2atm_); 2‐methyl‐hopanoid index = weighted average of the dominant 2‐methyl/2‐desmethylhopanoid pairs; *δ* ^13^C: carbon isotope values (in per mil vs. Vienna Pee Dee Belemnite) for bulk organic carbon (OC) and C_40:3cy_.

The abundance of ladderane fatty acids was investigated in sediments underlying the Arabian Sea OMZ, spanning the last 140 ka (Fig. 3a; Jaeschke *et al*., 2009). During this time period, the maxima in ladderane lipid concentration correlated with events of high total organic carbon (TOC) content and elevated *δ* ^15^N. This would appear to indicate enhanced anammox activity coincides with periods of increased intensity in the Arabian Sea OMZ and large‐scale changes in the nitrogen cycle (i.e. preferential removal of the lighter ^14^N isotope). Unfortunately, it has so far not been possible to assess anammox further back in time using ladderane lipids as they are not resistant to the degradative stresses of sediment burial. Ladderane fatty acids undergo β‐oxidation in the presence of even very low oxygen concentrations to form short chain ladderane fatty acids, which have been found in sediments underlying OMZs (Rush *et al*., 2011, 2012). However, these too appear to undergo further degradation, and these Arabian Sea sediments are currently the oldest known detection of past anammox activity (Jaeschke *et al*., 2009).

In order to potentially reveal degradation lipid products better‐suited for past detection of the anammox reaction, conditions undergone during catagenesis of organic matter were simulated; anammox biomass was artificially thermally matured via hydrous pyrolysis (Jaeschke *et al*., 2008). Ladderane lipids were found to be completely structurally altered at relatively low levels of thermal stress. However, thermally stable products in which some of the cyclobutane rings had opened, could still be detected at 260°C (Jaeschke *et al*., 2008). Additionally, unusual branched long chain alkanes originating from anammox biomass were identified in the oils generated during these artificial maturation experiments (Fig. 2; Rush *et al*., 2014b). Investigating these alkanes alongside BHT isomer might be an appropriate approach to detect anammox in thermally more mature sediments such as those deposited in the Cretaceous during so called oceanic anoxic events (OAEs).

## Heterocyst glycolipids: Expansion of cyanobacteria during arctic black shale deposition

Like the modern‐day Black Sea, the Arctic Ocean during the middle Eocene (ca. 49 Ma) was a warm, stratified basin with fresh surface waters. Such conditions induced an intense bloom of the free‐floating aquatic fern, *Azolla*. *Azolla* microspores occurred abundantly in the palynological record (Brinkhuis *et al*., 2006), and corresponded to increased TOC values. These *Azolla* ferns probably thrived in the fresh Arctic surface waters and high CO_2_ atmosphere. The carbon fixed by *Azolla* subsequently represented a significant sink of CO_2_ as their biomass was incorporated into buried sedimentary organic matter (Speelman *et al*., 2009). Bauersachs *et al*. (2010) found co‐variance in the enhanced input of *Azolla* spores and the abundance of heterocyst glycolipids in these high TOC Eocene Arctic black shale sediments (Fig. 3b). Additionally, the averaged values of the bulk sedimentary nitrogen isotope ratio (*δ* ^15^N) at the time were −1‰, which is consistent with a primarily diazotrophic signal. These results would seem to indicate that diazotrophs lived in symbiosis with *Azolla*, as seen in modern systems (Peters, 1991), and that they supplied fixed nitrogen to a phytoplankton community in the stratified Arctic Ocean. It is possible that N_2_‐fixation sustained photoautotrophic productivity that formed the black shales of the Eocene Arctic. These results demonstrate the excellent potential of heterocyst glycolipids as biomarkers for past diazotrophy.

## 2‐Methyl‐hopanoids: Cyanobacteria sustained primary production during cretaceous OAEs

Past expansions of OMZs have been hypothesised to trigger OAEs (Jenkyns, 2010). OAEs represent one of the most abrupt perturbations of major global biogeochemical cycles of the last 200 Ma, and were accompanied by a rise of the redoxcline to such an extent that sulphide‐containing waters occurred in the photic zone, providing a niche for photosynthetic anoxygenic green sulfur bacteria (Sinninghe Damsté and Köster, 1998). This resulted in widespread deposition of organic rich black shales (Arthur and Sageman, 1994). The extreme scenario of an OAE would involve a total reorganisation of the nitrogen cycle.

It is probable that like the Eocene Arctic, surface waters of Cretaceous OAEs 1a and 2 were characterized by increased cyanobacterial activity. The extraordinary abundance of, albeit less source‐specific, 2‐Me‐hopanoids during these OAEs (Fig. 3c) indicates a shift towards diazotrophy as the primary source of nitrogen (Kuypers *et al*., 2004). Increased N_2_‐fixation could have been induced by limited vertical mixing of deep‐water nitrogen species due to a stratification of the water column. It has been proposed that increased denitrification and anammox activity in the expanded anoxic waters of OAEs would have led to a substantial decrease in nitrogen availability (Kuypers *et al*., 2004). In order sustain oceanic primary productivity, this loss of nitrogen would have needed to be compensated by increased cyanobacterial N_2_‐fixation. This is seemingly apparent by the increased levels of 2‐Me hopanoids (Fig. 3c). However, this nitrogen loss hypothesis should still be tested by investigating BHT isomer and branched long chain alkanes in these OAE sediments.

## Crenarchaeol: Albian OAE bloom of thaumarchaeota

In disagreement with lipid evidence from OAEs 1a and 2, biomarkers for diazotrophs were not detected in Albian OAE 1b sediments from the proto North Atlantic and Tethys Ocean (Kuypers *et al*., 2001; Tsikos *et al*., 2004). In contrast, OAE 1b sediments contained an unprecedented contribution of crenarchaeol and organic material derived from nitrifying archaea (e.g. Fig. 3d; proto North Atlantic; Kuypers *et al*., 2001). This was evident from the *δ* ^13^C isotopic values of TOC, which was substantially enriched (∼−20‰) during OAE 1b, compared with non‐OAE conditions (∼−25‰) (Fig. 3d). Neither plant (−28 to −29‰; leaf‐waxes), algal (−27 to −29‰; steroids), nor bacterial (−24 to −27‰; hopanes) lipids showed the large change in *δ* ^13^C observed for TOC, suggesting a temporary source of ^13^C‐enriched organic matter during OAE 1b (Kuypers *et al*., 2002). Extant Thaumarchaeota use a modified hydroxypropionate‐hydroxybutyrate cycle carbon fixation pathway (Berg *et al*., 2010; Könneke *et al*., 2014), which results in ^13^C‐enriched AOA lipids in sediment of the present‐day ocean (Schouten *et al*., 2013b). In agreement with this, the tricyclic biphytane (C_40:3cy_), a derived ether‐cleaved component of crenarchaeol, is ^13^C‐enriched during OAE 1b (−17 to −18‰; Fig. 3). The same holds for AOA‐derived macromolecular matter (Kuypers *et al*., 2002). Thus, the ^13^C enrichment of bulk TOC in OAE 1b was likely due to enhanced archaeal contribution to the bulk organic carbon pool. These results would seem to indicate that AOA played a significant role in the onset and maintenance of OAE 1b.

The reason for the abundance of AOA during OAE 1b remains enigmatic. It seems that only a trigger independent of primary production (the source of organic nitrogen and ultimately ammonium in the present‐day ocean) could effect this change. The mid‐Cretaceous was a period of increased oceanic crust formation through enhanced volcanism (Larson, 1991). This volcanic activity would have introduced vast amounts of ${\text{NH}}_{4}^{+}$ to the marine water column. As some nitrifiers can function at very low oxygen concentrations, they can thrive at the oxic‐anoxic water boundary and provide oxidised nitrogen species to denitrifying organisms in anaerobic niches (Lam and Kuypers, 2011; Füssel *et al*., 2012). During OAE 1b, it is possible that AOA were oxidising volcanic ${\text{NH}}_{4}^{+}$ to ${\text{NO}}_{2}^{-}$, which was provided to denitrifying organisms. The consequent production of N_2_ before this bio‐available nitrogen could be exploited by photoautotrophs may have helped maintain water column anoxia during the Albian OAE. In comparison, injection of ${\text{NH}}_{4}^{+}$ from a modern submarine arc‐volcano has been shown to support a nitrifying archaea community (closely related to *Nitrosopumilus maritimus*) in Fe‐rich microbial mats (Kilias *et al*., 2013). Here, AOA is potentially supplying oxidised nitrogen species to be used to chemical oxidise hydrothermal Fe^2+^ into ferrihydrites.

These results show that there was no universal mechanism for OAE formation and black shale deposition in the Cretaceous. This further underscores the need to better constrain the marine nitrogen cycle during diverse past biogeochemical events in order to predict future change.

## Conclusions

Biomarker lipids have been used effectively to constrain past nitrogen cycling. However, available biomarkers are still limited, especially for anaerobic nitrogen cycling processes. Without biomarker observations in paleo‐environments, we cannot predict how the current expansion of marine oxygen minimum and anoxic zones will affect the removal of bio‐available nitrogen, nor the feedback mechanisms that will take place within the nitrogen cycle. Accordingly, more robust lipids must be developed as biomarkers for (anaerobic) nitrogen cycle processes.

In order for a biomarker to be used appropriately it must first be rigorously identified. Regrettably, this is often the most time consuming step in the pathway to introducing a novel biomarker. Advances in instrumentation (e.g. two‐dimensional nuclear magnetic resonance, X‐ray crystallography, scanning probe microscopy, gas chromatography, liquid chromatography, mass spectrometry) have primarily shaped the direction and limitation of biomarker identification/application. Often the biomarker community must wait for the development of improved methods in order to move forward with biomarker innovation. However, it is clearly essential to the advancement of novel biomarker for marine nitrogen cycle processes to invest the time in undertaking these types of investigations.

## References

[emi13682-bib-0001] Altieri, A.H. , and Gedan, K.B. (2015) Climate change and dead zones. Glob Change Biol 21: 1395–1406. 10.1111/gcb.12754 25385668

[emi13682-bib-0002] Arrigo, K.R. (2005) Marine microorganisms and global nutrient cycles. Nature 437: 349–355. 16163345 10.1038/nature04159

[emi13682-bib-0003] Arthur, M.A. , and Sageman, B.B. (1994) Marine black shales: depositional mechanisms and environments of ancient deposits. Annu Rev Earth Planet Sci 22: 499–551.

[emi13682-bib-0004] Auran, T.B. , and Schmidt, E.L. (1972) Similarities between *Hyphomicrobium* and *Nitrobacter* with respect to fatty acids. J Bacteriol 109: 450–451. 5057773 10.1128/jb.109.1.450-451.1972 PMC247301

[emi13682-bib-0005] Auran, T.B. , and Schmidt, E.L. (1976) Lipids of *Nitrobacter* and effects of cultural conditions on fatty‐acid composition. Biochim Biophys Acta 431: 390–398. 949483 10.1016/0005-2760(76)90205-8

[emi13682-bib-0006] Bale, N.J. , Hopmans, E.C. , Zell, C. , de Lima Sobrinho, R. , Kim, J.H. , Sinninghe Damsté, J.S. , *et al* (2015) Long chain glycolipids with pentose head groups as biomarkers for marine endosymbiotic heterocystous cyanobacteria. Org Geochem 81: 1–7.

[emi13682-bib-0007] Bauersachs, T. , Compaoré, J. , Hopmans, E.C. , Stal, L.J. , Schouten, S. , and Sinninghe Damsté, J.S. (2009a) Distribution of heterocyst glycolipids in cyanobacteria. Phytochemistry 70: 2034–2039. 19772975 10.1016/j.phytochem.2009.08.014

[emi13682-bib-0008] Bauersachs, T. , Hopmans, E.C. , Compaoré, J. , Stal, L.J. , Schouten, S. , and Sinninghe Damsté, J.S. (2009b) Rapid analysis of long‐chain glycolipids in heterocystous cyanobacteria using high‐performance liquid chromatography coupled to electrospray ionization tandem mass spectrometry. Rapid Commun Mass Spectrom 23: 1387–1394. 19347866 10.1002/rcm.4009

[emi13682-bib-0009] Bauersachs, T. , Speelman, E.N. , Hopmans, E.C. , Reichart, G.J. , Schouten, S. , and Sinninghe Damsté, J.S. (2010) Fossilized glycolipids reveal past oceanic N_2_ fixation by heterocystous cyanobacteria. Proc Natl Acad Sci U S A 107: 19190–19194. 20966349 10.1073/pnas.1007526107 PMC2984197

[emi13682-bib-0010] Bauersachs, T. , Mudimu, O. , Schulz, S. , and Schwark, L. (2014) Distribution of long chain heterocyst glycolipids in N_2_‐fixing cyanobacteria of the order Stigonematales. Phytochemistry 98: 145–150. 24361292 10.1016/j.phytochem.2013.11.007

[emi13682-bib-0011] Berg, I.A. , Kockelkorn, D. , Ramos‐Vera, W.H. , Say, R.F. , Zarzycki, J. , Hügler, M. , *et al* (2010) Autotrophic carbon fixation in archaea. Nat Rev Microbiol 8: 447–460. 20453874 10.1038/nrmicro2365

[emi13682-bib-0012] Bisseret, P. , Zundel, M. , and Rohmer, M. (1985) Prokaryotic Triterpenoids. 2. 2β‐methylhopanoids from *Methylobacterium organophilum* and *Nostoc muscorum*, a new series of prokaryotic triterpenoids. Eur J Biochem 150: 29–34. 3926495 10.1111/j.1432-1033.1985.tb08982.x

[emi13682-bib-0013] Bravo, J.M. , Perzl, M. , Härtner, T. , Kannenberg, E.L. , and Rohmer, M. (2001) Novel methylated triterpenoids of the gammacerane series from the nitrogen‐fixing bacterium *Bradyrhizobium japonicum* USDA 110. Eur J Biochem 268: 1323–1331. 11231284 10.1046/j.1432-1327.2001.01998.x

[emi13682-bib-0014] Brinkhuis, H. , Schouten, S. , Collinson, M.E. , Sluijs, A. , Sinninghe Damsté, J.S. , Dickens, G.R. , *et al* (2006) Episodic fresh surface waters in the Eocene Arctic Ocean. Nature 441: 606–609. 16752440 10.1038/nature04692

[emi13682-bib-1014] Brochier‐Armanet, C. , Boussau, B. , Gribaldo, S. , and Forterre, P. . (2008) Mesophilic crenarchaeota: proposal for a third archaeal phylum, the Thaumarchaeota. Nature Rev Microbiol 6: 245–252. 18274537 10.1038/nrmicro1852

[emi13682-bib-0015] Bryce, T.A. , Welti, D. , Walsby, A.E. , and Nichols, B.W. (1972) Monohexoside derivatives of long‐chain polyhydroxy alcohols: a novel class of glycolipid specific to heterocystous algae. Phytochemistry 11: 295.

[emi13682-bib-0016] Capone, D.G. , Zehr, J.P. , Pearl, H.W. , Bergman, B. , and Carpenter, E.J. (1997) *Trichodesmium*, a globally significant marine cyanobacterium. Science 276: 1221–1229.

[emi13682-bib-0017] Capone, D.G. (2001) Marine nitrogen fixation: what's the fuss? Curr Opin Microbiol 4: 341–348. 11378490 10.1016/s1369-5274(00)00215-0

[emi13682-bib-0018] Capone, D.G. (2008) The marine nitrogen cycle. Microbe 3: 186–192.

[emi13682-bib-0019] Carpenter, E.J. , and Romans, K. (1991) Major role of the cyanobacterium *Trichodesmium* in nutrient cycling in the North‐Atlantic Ocean. Science 254: 1356–1358. 17773605 10.1126/science.254.5036.1356

[emi13682-bib-0020] Carpenter, E.J. , Montoya, J.P. , Burns, J. , Mulholland, M.R. , Subramaniam, A. , and Capone, D.G. (1999) Extensive bloom of a N_2_‐fixing diatom/cyanobacterial association in the tropical Atlantic Ocean. Mar Ecol Prog Ser 185: 273–283.

[emi13682-bib-0021] Codispoti, L.A. , Brandes, J.A. , Christensen, J.P. , Devol, A.H. , Naqvi, S.W.A. , Paerl, H.W. , and Yoshinari, T. (2001) The oceanic fixed nitrogen and nitrous oxide budgets: moving targets as we enter the Anthropocene? Sci Mar 65: 85–105.

[emi13682-bib-0022] Cvejic, J.H. , Putra, S.R. , El‐Beltagy, A. , Hattori, R. , Hattori, T. , and Rohmer, M. (2000) Bacterial triterpenoids of the hopane series as biomarkers for the chemotaxonomy of *Burkholderia*, *Pseudomonas* and *Ralstonia* spp. FEMS Microbiol Lett 183: 295–299. 10675600 10.1111/j.1574-6968.2000.tb08974.x

[emi13682-bib-0023] Daims, H. , Nielsen, J.L. , Nielsen, P.H. , Schleifer, K.H. , and Wagner, M. (2001) In situ characterization of *Nitrospira*‐like nitrite‐oxidizing bacteria active in wastewater treatment plants. Appl Environ Microbiol 67: 5273–5284. 11679356 10.1128/AEM.67.11.5273-5284.2001 PMC93301

[emi13682-bib-0024] Daims, H. , Lebedeva, E.V. , Pjevac, P. , Han, P. , Herbold, C. , Albertsen, M. , *et al* (2015) Complete nitrification by *Nitrospira* bacteria. Nature 528: 504–509. 26610024 10.1038/nature16461 PMC5152751

[emi13682-bib-0025] Dalsgaard, T. , and Thamdrup, B. (2002) Factors controlling anaerobic ammonium oxidation with nitrite in marine sediments. Appl Environ Microbiol 68: 3802–3808. 12147475 10.1128/AEM.68.8.3802-3808.2002 PMC124030

[emi13682-bib-0026] Dalsgaard, T. , Thamdrup, B. , Farias, L. , and Revsbech, N.P. (2012) Anammox and denitrification in the oxygen minimum zone of the eastern South Pacific. Limnol Oceanogr 57: 1331–1346.

[emi13682-bib-0027] De Brabandere, L. , Canfield, D.E. , Dalsgaard, T. , Friederich, G.E. , Revsbech, N.P. , Ulloa, O. , and Thamdrup, B. (2014) Vertical partitioning of nitrogen‐loss processes across the oxic‐anoxic interface of an oceanic oxygen minimum zone. Environ Microbiol 16: 3041–3054. 24118779 10.1111/1462-2920.12255

[emi13682-bib-0028] Dore, J.E. , Brum, J.R. , Tupas, L.M. , and Karl, D.M. (2002) Seasonal and interannual variability in sources of nitrogen supporting export in the oligotrophic subtropical North Pacific Ocean. Limnol Oceanogr 47: 1595–1607.

[emi13682-bib-0029] Ettwig, K.F. , Butler, M.K. , Le Paslier, D. , Pelletier, E. , Mangenot, S. , Kuypers, M.M.M. , *et al* (2010) Nitrite‐driven anaerobic methane oxidation by oxygenic bacteria. Nature 464: 543. U94. 20336137 10.1038/nature08883

[emi13682-bib-0030] Farnelid, H. , Andersson, A.F. , Bertilsson, S. , Al‐Soud, W.A. , Hansen, L.H. , Sørensen, S. , *et al* (2011) Nitrogenase gene amplicons from global marine surface waters are dominated by genes of non‐cyanobacteria. PLoS One 6: e19223. 21559425 10.1371/journal.pone.0019223 PMC3084785

[emi13682-bib-0031] Farrimond, P. , Fox, P.A. , Innes, H.E. , Miskin, I.P. , and Head, I.M. (1998) Bacterial source of hopanoids in Recent sediments: improving our understanding of ancient hopane biomarkers. Anc Biomol 2: 147–166.

[emi13682-bib-0032] Francis, C.A. , Roberts, K.J. , Beman, J.M. , Santoro, A.E. , and Oakley, B.B. (2005) Ubiquity and diversity of ammonia‐oxidizing archaea in water columns and sediments of the ocean. Proc Natl Acd Sci U S A 102: 14683–14688. 10.1073/pnas.0506625102 PMC1253578 16186488

[emi13682-bib-0033] Füssel, J. , Lam, P. , Lavik, G. , Jensen, M.M. , Holtappels, M. , Günter, M. , and Kuypers, M.M.M. (2012) Nitrite oxidation in the Namibian oxygen minimum zone. ISME J 6: 1200–1209. 22170426 10.1038/ismej.2011.178 PMC3358024

[emi13682-bib-0034] Gallon, J.R. (2001) N_2_ fixation in phototrophs: adaptation to a specialized way of life. Plant Soil 230: 39–48.

[emi13682-bib-0035] Gruber, N. (2004) The dynamics of the marine nitrogen cycle and its influence on atmospheric CO_2_ variations In Carbon Climate Interactions. Oguz T. (ed). Hoboken, NY: John Wiley, pp. 97–148.

[emi13682-bib-0036] Gruber, N. (2008) The marine nitrogen cycle: overview and challenges In Nitrogen in the marine environment. Capone, D.G. , Bronk, D.A. , Mulholland, M.R. , and Carpenter, E.J. (eds). Burlington, MA: Elsevier, pp. 1–50.

[emi13682-bib-0037] Hallam, S.J. , Mincer, T.J. , Schleper, C. , Preston, C.M. , Roberts, K. , Richardson, P.M. , and DeLong, E.F. (2006) Pathways of carbon assimilation and ammonia oxidation suggested by environmental genomic analyses of marine Crenarchaeota. Plos Biol 4: 520–536. 10.1371/journal.pbio.0040095 PMC1403158 16533068

[emi13682-bib-0038] Hamersley, M.R. , Lavik, G. , Woebken, D. , Rattray, J.E. , Lam, P. , Hopmans, E.C. , *et al* (2007) Anaerobic ammonium oxidation in the Peruvian oxygen minimum zone. Limnol Oceanogr 52: 923–933.

[emi13682-bib-0039] Handley, L. , Talbot, H.M. , Cooke, M.P. , Anderson, K.E. , and Wagner, T. (2010) Bacteriohopanepolyols as tracers for continental and marine organic matter supply and phases of enhanced nitrogen cycling on the late Quaternary Congo deep sea fan. Org Geochem 41: 910–914.

[emi13682-bib-0040] Herrmann, D. , Bisseret, P. , Connan, J. , and Rohmer, M. (1996) A non‐extractable triterpenoid of the hopane series in *Acetobacter xylinum* . FEMS Microbiol Lett 135: 323–326.

[emi13682-bib-0041] Ho, S.L. , and Laepple, T. (2016) Flat meridional temperature gradient in the early Eocene in the subsurface rather than surface ocean. Nat Geosci 9: 606–610.

[emi13682-bib-0042] Hofreiter, M. , Serre, D. , Poinar, H.N. , Kuch, M. , and Pääbo, S. (2001) Ancient DNA. Nat Rev Genet 2: 353–359. 11331901 10.1038/35072071

[emi13682-bib-0043] Hu, A. , Jiao, N. , Zhang, R. , and Yang, Z. (2011) Niche partitioning of Marine Group I Crenarchaeota in the euphotic and upper mesopelagic zones of the East China Sea. Appl Environ Microbiol 77: 7469–7478. 21873485 10.1128/AEM.00294-11 PMC3209141

[emi13682-bib-0044] Hurley, S.J. , Elling, F.J. , Könneke, M. , Buchwald, C. , Wankel, S.D. , Santoro, A.E. , *et al* (2016) Influence of ammonia oxidation rate on thaumarchaeal lipid composition and the TEX_86_ temperature proxy. Proc Natl Acad Sci U S A 113: 7762–7767. 27357675 10.1073/pnas.1518534113 PMC4948339

[emi13682-bib-0045] Jaeschke, A. , Lewan, M.D. , Hopmans, E.C. , Schouten, S. , and Sinninghe Damsté, J.S. (2008) Thermal stability of ladderane lipids as determined by hydrous pyrolysis. Org Geochem 39: 1735–1741.

[emi13682-bib-0046] Jaeschke, A. , Ziegler, M. , Hopmans, E.C. , Reichart, G.J. , Lourens, L.J. , Schouten, S. , and Sinninghe Damsté, J.S. (2009) Molecular fossil evidence for anaerobic ammonium oxidation in the Arabian Sea over the last glacial cycle. Paleoceanography 24: PA2202‐1–PA2202‐11.

[emi13682-bib-0047] Jenkyns, H.C. (2010) Geochemistry of oceanic anoxic events. *Geochem* . Geophys Geosystems 11: 1–30.

[emi13682-bib-0048] Jenkyns, H.C. , Schouten‐Huibers, L. , Schouten, S. , and Sinninghe Damsté, J.S. (2012) Warm middle Jurassic‐Early Cretaceous high‐latitude sea‐surface temperatures from the Southern Ocean. Clim Past 8: 215–226.

[emi13682-bib-0049] Karl, D. , Letelier, R. , Tupas, L. , Dore, J. , Christian, J. , and Hebel, D. (1997) The role of nitrogen fixation in biogeochemical cycling in the subtropical North Pacific Ocean. Nature 388: 533–538.

[emi13682-bib-0050] Karner, M.B. , DeLong, E.F. , and Karl, D.M. (2001) Archaeal dominance in the mesopelagic zone of the Pacific Ocean. Nature 409: 507–510. 11206545 10.1038/35054051

[emi13682-bib-0051] van Kessel, M.A.H.J. , Speth, D.R. , Albertsen, M. , Nielsen, P.H. , Op den Camp, H.J.M. , Kartal, B. , *et al* (2015) Complete nitrification by a single microorganism. Nature 528: 555–559. 26610025 10.1038/nature16459 PMC4878690

[emi13682-bib-0052] Kilias, S.P. , Nomikou, P. , Papanikolaou, D. , Polymenakou, P.N. , Godelitsas, A. , Argyraki, A. , *et al* (2013) New insights into hydrothermal vent processes in the unique shallow‐submarine arc‐volcano, Kolumbo (Santorini), Greece. Sci Rep 3: 2421. 23939372 10.1038/srep02421 PMC3741630

[emi13682-bib-0053] Kim, J.H. , van der Meer, J. , Schouten, S. , Helmke, P. , Willmott, V. , Sangiorgi, F. , *et al* (2010) New indices and calibrations derived from the distribution of crenarchaeal isoprenoid tetraether lipids: implications for past sea surface temperature reconstructions. Geochim Cosmochim Acta 74: 4639–4654.

[emi13682-bib-0054] Kim, J.H. , Schouten, S. , Rodrigo‐Gamiz, M. , Rampen, S. , Marino, G. , Huguet, C. , *et al* (2016) Influence of deep‐water derived isoprenoid tetraether lipids on the paleothermometer in the Mediterranean Sea. Geochim Cosmochim Acta 150: 125–141.

[emi13682-bib-0055] Knani, M. , Corpe, W.A. , and Rohmer, M. (1994) Bacterial hopanoids from pink‐pigmented facultative methylotrophs (PPFMs) and from green plant surfaces. Microbiology 140: 2755–2759.

[emi13682-bib-0056] Könneke, M. , Bernhard, A.E. , de la Torre, J.R. , Walker, C.B. , Waterbury, J.B. , and Stahl, D.A. (2005) Isolation of an autotrophic ammonia‐oxidizing marine archaeon. Nature 437: 543–546. 16177789 10.1038/nature03911

[emi13682-bib-0057] Könneke, M. , Schubert, D.M. , Brown, P.C. , Hügler, M. , Standfest, S. , Schwander, T. , *et al* (2014) Ammonia‐oxidizing archaea use the most energy‐efficient aerobic pathway for CO_2_ fixation. Proc Natl Acad Sci 111: 8239–8244. 24843170 10.1073/pnas.1402028111 PMC4050595

[emi13682-bib-0058] Kool, D.M. , Talbot, H.M. , Rush, D. , Ettwig, K. , and Sinninghe Damsté, J.S. (2014) Rare bacteriohopanepolyols as markers for an autotrophic, intra‐aerobic methanotroph. Geochim Cosmochim Acta 136: 114–125.

[emi13682-bib-0059] Kuypers, M.M.M. , Blokker, P. , Erbacher, J. , Kinkel, H. , Pancost, R.D. , Schouten, S. , and Sinninghe Damsté, J.S. (2001) Massive expansion of marine archaea during a mid‐Cretaceous oceanic anoxic event. Science 293: 92–94. 11441180 10.1126/science.1058424

[emi13682-bib-0060] Kuypers, M.M.M. , Blokker, P. , Hopmans, E.C. , Kinkel, H. , Pancost, R.D. , Schouten, S. , and Sinninghe Damsté, J.S. (2002) Archaeal remains dominate marine organic matter from the early Albian oceanic anoxic event 1b. Palaeogeogr Palaeoclimatol Palaeoecol 185: 211–234.

[emi13682-bib-0061] Kuypers, M.M.M. , Sliekers, A.O. , Lavik, G. , Schmid, M. , Jørgensen, B.B. , Kuenen, J.G. , *et al* (2003) Anaerobic ammonium oxidation by anammox bacteria in the Black Sea. Nature 422: 608–611. 12686999 10.1038/nature01472

[emi13682-bib-0062] Kuypers, M.M.M. , van Breugel, Y. , Schouten, S. , Erba, E. , and Sinninghe Damsté, J.S. (2004) N_2_‐fixing cyanobacteria supplied nutrient N for Cretaceous oceanic anoxic events. Geology 32: 853–856.

[emi13682-bib-0063] Kuypers, M.M.M. , Lavik, G. , Woebken, D. , Schmid, M. , Fuchs, B.M. , Amann, R. , *et al* (2005) Massive nitrogen loss from the Benguela upwelling system through anaerobic ammonium oxidation. Proc Natl Acad Sci U S A 102: 6478–6483. 15843458 10.1073/pnas.0502088102 PMC556276

[emi13682-bib-0064] Lam, P. , Lavik, G. , Jensen, M.M. , van de Vossenberg, J. , Schmid, M. , Woebken, D. , *et al* (2009) Revising the nitrogen cycle in the Peruvian oxygen minimum zone. Proc Natl Acad Sci U S A 106: 4752–4757. 19255441 10.1073/pnas.0812444106 PMC2649953

[emi13682-bib-0065] Lam, P. , and Kuypers, M.M.M. (2011) Microbial nitrogen cycling processes in oxygen minimum zones Ann Rev Mar Sci 3: 317–345. 10.1146/annurev-marine-120709-142814 21329208

[emi13682-bib-0066] Lambein, F. , and Wolk, C.P. (1973) Structural studies on glycolipids from envelope of heterocyst of *Anabaena cylindrica* . Biochemistry (Mosc.) 12: 791–798. 10.1021/bi00729a002 4631370

[emi13682-bib-0067] Larson, R.L. (1991) Latest pulse of Earth: evidence for a mid‐Cretaceous superplume. Geology 19: 547–550.

[emi13682-bib-0068] Lavin, M.F. , Fiedler, P.C. , Amador, J.A. , Ballance, L.T. , Färber‐Lorda, J. , and Mestas‐Nunez, A.M. (2006) A review of eastern tropical Pacific oceanography: summary. Prog Oceanogr 69: 391–398.

[emi13682-bib-0069] Lebedeva, E.V. , Off, S. , Zumbrägel, S. , Kruse, M. , Shagzhina, A. , Lücker, S. , *et al* (2011) Isolation and characterization of a moderately thermophilic nitrite‐oxidizing bacterium from a geothermal spring. FEMS Microbiol Ecol 75: 195–204. 21138449 10.1111/j.1574-6941.2010.01006.x

[emi13682-bib-0070] Lincoln, S.A. , Wai, B. , Eppley, J.M. , Church, M.J. , Summons, R.E. , and DeLong, E.F. (2014) Planktonic Euryarchaeota are a significant source of archaeal tetraether lipids in the ocean. Proc Natl Acad Sci U S A 111: 9858–9863. 24946804 10.1073/pnas.1409439111 PMC4103328

[emi13682-bib-0071] Lipski, A. , Spieck, E. , Makolla, A. , and Altendorf, K. (2001) Fatty acid profiles of nitrite‐oxidizing bacteria reflect their phylogenetic heterogeneity. Syst Appl Microbiol 24: 377–384. 11822673 10.1078/0723-2020-00049

[emi13682-bib-0072] Lücker, S. , Nowka, B. , Rattei, T. , Spieck, E. , and Daims, H. (2013) The genome of *Nitrospina gracilis* illuminates the metabolism and evolution of the major marine nitrite oxidizer. Front Microbiol 4: 1–19. 23439773 10.3389/fmicb.2013.00027 PMC3578206

[emi13682-bib-0073] Mahaffey, C. , Michaels, A.F. , and Capone, D.G. (2005) The conundrum of marine N_2_ fixation. Am J Sci 305: 546–595.

[emi13682-bib-0074] Martínez‐Pérez, C. , Mohr, W. , Löscher, C.R. , Dekaezemacker, J. , Littmann, S. , Yilmaz, P. , *et al* (2016) The small unicellular diazotrophic symbiont, UCYN‐A, is a key player in the marine nitrogen cycle. Nat Microbiol 1: 16163. 27617976 10.1038/nmicrobiol.2016.163

[emi13682-bib-0075] Mascitti, V. , and Corey, E.J. (2004) Total synthesis of (+/‐)‐pentacycloanammoxic acid. J Am Chem Soc 126: 15664–15665. 15571387 10.1021/ja044089a

[emi13682-bib-0076] Mercer, J.A. , Cohen, C.M. , Shuken, S.R. , Wagner, A.M. , Smith, M.W. , Moss, F.R., III ., *et al* (2016) Chemical Synthesis and Self‐Assembly of a Ladderane Phospholipid. J Am Chem Soc 138: 15845–15848. 27960308 10.1021/jacs.6b10706 PMC5279923

[emi13682-bib-0077] Monteiro, F.M. , Pancost, R.D. , Ridgwell, A. , and Donnadieu, Y. (2012) Nutrients as the dominant control on the spread of anoxia and euxinia across the Cenomanian‐Turonian oceanic anoxic event (OAE2): model‐data comparison. Paleoceanography 27: PA4209.

[emi13682-bib-0078] Mulder, A. , van de Graaf, A.A. , Robertson, L.A. , and Kuenen, J.G. (1995) Anaerobic ammonium oxidation discovered in a denitrifying fluidized‐bed reactor. FEMS Microbiol Ecol 16: 177–183.

[emi13682-bib-0079] Murry, M.A. , and Wolk, C.P. (1989) Evidence that the barrier to the penetration of oxygen into heterocysts depends upon two layers of the cell envelope. Arch Microbiol 151: 469–474.

[emi13682-bib-0080] Mußmann, M. , Brito, I. , Pitcher, A. , Sinninghe Damsté, J.S.S. , Hatzenpichler, R. , Richter, A. , *et al* (2011) Thaumarchaeotes abundant in refinery nitrifying sludges express amoA but are not obligate autotrophic ammonia oxidizers. Proc Natl Acad Sci U S A 108: 16771–16776. 21930919 10.1073/pnas.1106427108 PMC3189051

[emi13682-bib-0081] Neumann, S. , Wessels, H.J.C.T. , Rijpstra, W.I.C. , Sinninghe Damsté, J.S. , Kartal, B. , Jetten, M.S.M. , and van Niftrik, L. (2014) Isolation and characterization of a prokaryotic cell organelle from the anammox bacterium *Kuenenia stuttgartiensis* . Mol Microbiol 94: 794–802. 25287816 10.1111/mmi.12816

[emi13682-bib-0082] Nichols, B.W. , and Wood, B.J.B. (1968) New glycolipid specific to nitrogen‐fixing blue‐green algae. Nature 217: 767.

[emi13682-bib-0083] Ourisson, G. , and Albrecht, P. (1992) Hopanoids. 1. Geohopanoids: the most abundant natural products on earth? Acc Chem Res 25: 398–402.

[emi13682-bib-0084] Padilla, C.C. , Bristow, L.A. , Sarode, N. , Garcia‐Robledo, E. , Gomez Ramirez, E. , Benson, C.R. , *et al* (2016) NC10 bacteria in marine oxygen minimum zones. ISME J 10: 2067–2071. 26918666 10.1038/ismej.2015.262 PMC5029155

[emi13682-bib-0085] Paulmier, A. , and Ruiz‐Pino, D. (2009) Oxygen minimum zones (OMZs) in the modern ocean. Prog Oceanogr 80: 113–128.

[emi13682-bib-0086] Pester, M. , Maixner, F. , Berry, D. , Rattei, T. , Koch, H. , Lücker, S. , *et al* (2014) NxrB encoding the beta subunit of nitrite oxidoreductase as functional and phylogenetic marker for nitrite‐oxidizing *Nitrospira* . Environ Microbiol 16: 3055–3071. 24118804 10.1111/1462-2920.12300

[emi13682-bib-0087] Peters, G.A. (1991) *Azolla* and other plant‐cyanobacteria symbioses: aspects of form and function. Plant Soil 137: 25–36.

[emi13682-bib-0088] Peters, K.E. , Walters, C.C. , and Moldowan, J.M. (2005) The Biomarker Guide: Biomarkers and Isotopes in Petroleum Exploration and Earth History, 2nd ed New York, Cambridge University Press.

[emi13682-bib-0089] Pinto, A.J. , Marcus, D.N. , Ijaz, U.Z. , Santos, Q.M.B. , Lose, Dick, G.J. , and Raskin, L. (2016) Metagenomic evidence for the presence of comammox *Nitrospira*‐like bacteria in a drinking water system. mSphere 1: e00054‐15. 27303675 10.1128/mSphere.00054-15 PMC4863621

[emi13682-bib-0090] Pitcher, A. , Villanueva, L. , Hopmans, E.C. , Schouten, S. , and Sinninghe Damsté, J.S. (2011) Niche segregation of ammonia‐oxidizing archaea and anammox bacteria in the Arabian Sea oxygen minimum zone. ISME J 5: 1896–1904. 21593795 10.1038/ismej.2011.60 PMC3223301

[emi13682-bib-0091] Rabotyagov, S.S. , Kling, C.L. , Gassman, P.W. , Rabalais, N.N. , and Turner, R.E. (2014) The economics of dead zones: causes, impacts, policy challenges, and a model of the Gulf of Mexico hypoxic zone. Rev Environ Econ Policy 8: 58–79.

[emi13682-bib-0092] Rashby, S.E. , Sessions, A.L. , Summons, R.E. , and Newman, D.K. (2007) Biosynthesis of 2‐methylbacteriohopanepolyols by an anoxygenic phototroph. Proc Natl Acad Sci 104: 15099–15104. 17848515 10.1073/pnas.0704912104 PMC1986619

[emi13682-bib-0093] Risgaard‐Petersen, N. , Langezaal, A.M. , Ingvardsen, S. , Schmid, M.C. , Jetten, M.S.M. , Op den Camp, H.J.M. , *et al* (2006) Evidence for complete denitrification in a benthic foraminifer. Nature 443: 93–96. 16957731 10.1038/nature05070

[emi13682-bib-0094] Rush, D. , Jaeschke, A. , Hopmans, E.C. , Geenevasen, J.A.J. , Schouten, S. , and Sinninghe Damsté, J.S. (2011) Short chain ladderanes: oxic biodegradation products of anammox lipids. Geochim Cosmochim Acta 75: 1662–1671.

[emi13682-bib-0095] Rush, D. , Hopmans, E.C. , Wakeham, S.G. , Schouten, S. , and Sinninghe Damsté, J.S. (2012) Occurrence and distribution of ladderane oxidation products in different oceanic regimes. Biogeosciences 9: 2407–2418.

[emi13682-bib-0096] Rush, D. , Sinninghe Damsté, J.S. , Poulton, S.W. , Thamdrup, B. , Garside, A. , Gonzalez, J.A. , *et al* (2014a) Anaerobic ammonium‐oxidising bacteria: a biological source of the bacteriohopanetetrol stereoisomer in marine sediments. Geochim Cosmochim Acta 140: 50–64.

[emi13682-bib-0097] Rush, D. , Jaeschke, A. , Geenevasen, J.A. , Tegelaar, E. , Pureveen, J. , Lewan, M.D. , *et al* (2014b) Generation of unusual branched long chain alkanes from hydrous pyrolysis of anammox bacterial biomass. Org Geochem 76: 136–145.

[emi13682-bib-0098] Sakata, S. , Hayes, J.M. , Rohmer, M. , Hooper, A.B. , and Seemann, M. (2008) Stable carbon‐isotopic compositions of lipids isolated from the ammonia‐oxidizing chemoautotroph *Nitrosomonas europaea* . Org Geochem 39: 1725–1734.

[emi13682-bib-0099] Schouten, S. , Hopmans, E.C. , Schefuß, E. , and Sinninghe Damsté, J.S. (2002) Distributional variations in marine crenarchaeotal membrane lipids: a new tool for reconstructing ancient sea water temperatures? Earth Planet Sci Lett 204: 265–274.

[emi13682-bib-0100] Schouten, S. , Pitcher, A. , Hopmans, E.C. , Villanueva, L. , van Bleijswijk, J. , and Sinninghe Damsté, J.S. (2012) Intact polar and core glycerol dibiphytanyl glycerol tetraether lipids in the Arabian Sea oxygen minimum zone: I. Selective preservation and degradation in the water column and consequences for the TEX_86_ . Geochim Cosmochim Acta 98: 228–243.

[emi13682-bib-0101] Schouten, S. , Villareal, T.A. , Hopmans, E.C. , Mets, A. , Swanson, K.M. , and Sinninghe Damsté, J.S. (2013a) Endosymbiotic heterocystous cyanobacteria synthesize different heterocyst glycolipids than free‐living heterocystous cyanobacteria. Phytochemistry 85: 115–121. 23044080 10.1016/j.phytochem.2012.09.002

[emi13682-bib-0102] Schouten, S. , Hopmans, E.C. , and Sinninghe Damsté, J.S. (2013b) The organic geochemistry of glycerol dialkyl glycerol tetraether lipids: a review. Org Geochem 54: 19–61.

[emi13682-bib-0103] Schouten, S. , Villanueva, L. , Hopmans, E.C. , van der Meer, M.T.J. , and Damste, J.S.S. (2014) Are Marine Group II Euryarchaeota significant contributors to tetraether lipids in the ocean?. Proc Natl Acad Sci U S A 111: E4285–E4285. 25239232 10.1073/pnas.1416176111 PMC4205633

[emi13682-bib-0104] Shah, S.R. , Mollenhauer, G. , Ohkouchi, N. , Eglinton, T.I. , and Pearson, A. (2008) Origins of archaeal tetraether lipids in sediments: insights from radiocarbon analysis. Geochim Cosmochim Acta 72: 4577–4594.

[emi13682-bib-0105] Simonin, P. , Tindall, B. , and Rohmer, M. (1994) Structure elucidation and biosynthesis of 31‐methylhopanoids from *Acetobacter europaeus* . Eur J Biochem 225: 765–771. 7957191 10.1111/j.1432-1033.1994.00765.x

[emi13682-bib-0106] Sinninghe Damsté, J.S. , and Köster, J. (1998) A euxinic southern North Atlantic Ocean during the Cenomanian/Turonian oceanic anoxic event. Earth Planet. Sci Lett 158: 165–173.

[emi13682-bib-0107] Sinninghe Damsté, J.S. , Schouten, S. , Hopmans, E.C. , van Duin, A.C.T. , and Geenevasen, J.A.J. (2002a) Crenarchaeol: the characteristic core glycerol dibiphytanyl glycerol tetraether membrane lipid of cosmopolitan pelagic Crenarchaeota. J Lipid Res 43: 1641–1651. 12364548 10.1194/jlr.m200148-jlr200

[emi13682-bib-0108] Sinninghe Damsté, J.S. , Strous, M. , Rijpstra, W.I.C. , Hopmans, E.C. , Geenevasen, J.A.J. , van Duin, A.C.T. , *et al* (2002b) Linearly concatenated cyclobutane lipids form a dense bacterial membrane. Nature 419: 708–712. 12384695 10.1038/nature01128

[emi13682-bib-0109] Sollai, M. , Hopmans, E.C. , Schouten, S. , Keil, R.G. , and Sinninghe Damsté, J.S. (2015) Intact polar lipids of Thaumarchaeota and anammox bacteria as indicators of N cycling in the eastern tropical North Pacific oxygen‐deficient zone. Biogeosciences 12: 4725–4737.

[emi13682-bib-1110] Spang, A. , Hatzenpichler, R. , Brochier‐Armanet, C. , Rattei, T. , Tischler, P. , Spieck, E. , *et al* (2010) Distinct gene set in two different lineages of ammonia‐oxidizing archaea supports the phylum Thaumarchaeota. Trends Microbiol 18: 331–340. 20598889 10.1016/j.tim.2010.06.003

[emi13682-bib-0110] Speelman, E.N. , van Kempen, M.M.L. , Barke, J. , Brinkhuis, H. , Reichart, G.J. , Smolders, A.J.P. , *et al* (2009) The Eocene Arctic *Azolla* bloom: environmental conditions, productivity and carbon drawdown. Geobiology 7: 155–170. 19323694 10.1111/j.1472-4669.2009.00195.x

[emi13682-bib-0111] Strous, M. , Fuerst, J.A. , Kramer, E.H.M. , Logemann, S. , Muyzer, G. , van de Pas‐Schoonen, K.T. , *et al* (1999) Missing lithotroph identified as new Planctomycete. Nature 400: 446–449. 10440372 10.1038/22749

[emi13682-bib-0112] Summons, R.E. , Jahnke, L.L. , Hope, J.M. , and Logan, G.A. (1999) 2‐Methylhopanoids as biomarkers for cyanobacterial oxygenic photosynthesis. Nature 400: 554–557. 10448856 10.1038/23005

[emi13682-bib-0113] Talbot, H.M. , and Farrimond, P. (2007) Bacterial populations recorded in diverse sedimentary biohopanoid distributions. Org Geochem 38: 1212–1225.

[emi13682-bib-0114] Talbot, H.M. , Rohmer, M. , and Farrimond, P. (2007) Structural characterisation of unsaturated bacterial hopanoids by atmospheric pressure chemical ionisation liquid chromatography/ion trap mass spectrometry. Rapid Commun Mass Spectrom 21: 1613–1622. 17443490 10.1002/rcm.2997

[emi13682-bib-0115] Talbot, H.M. , Summons, R.E. , Jahnke, L.L. , Cockell, C.S. , Rohmer, M. , and Farrimond, P. (2008) Cyanobacterial bacteriohopanepolyol signatures from cultures and natural environmental settings. Org Geochem 39: 232–263.

[emi13682-bib-0116] Talbot, H.M. , Bischoff, J. , Inglis, G.N. , Collinson, M.E. , and Pancost, R.D. (2016) Polyfunctionalised bio‐ and geohopanoids in the Eocene Cobham Lignite. Org Geochem 96: 77–92.

[emi13682-bib-0117] Tsikos, H. , Karakitsios, V. , van Breugel, Y. , Walsworth‐Bell, B. , Bombardiere, L. , Petrizzo, M.R. , *et al* (2004) Organic‐carbon deposition in the Cretaceous of the Ionian basin, NW Greece: the Paquier Event (OAE 1b) revisited. Geol Mag 141: 401–416.

[emi13682-bib-0118] Villanueva, L. , Schouten, S. , and Sinninghe Damsté, J.S. (2015) Depth‐related distribution of a key gene of the tetraether lipid biosynthetic pathway in marine Thaumarchaeota. Environ Microbiol 17: 3527–3539. 24813867 10.1111/1462-2920.12508

[emi13682-bib-0119] Villanueva, L. , Schouten, S. , and Damsté, J.S.S. (2017) Phylogenomic analysis of lipid biosynthetic genes of Archaea shed light on the “lipid divide”. Environ Microbiol 19: 54–69. 27112361 10.1111/1462-2920.13361

[emi13682-bib-0120] Villareal, T.A. (1992) Marine nitrogen‐fixing diatom‐cyanobacteria symbioses In Marine Pelagic Cyanobacteria: Trichodesmium and Other Diazotrophs. Carpenter E.J., Capone D.G., and Rueter J.G (eds) The Netherlands: Kluwer Academic, Dordrecht, pp. 163–175.

[emi13682-bib-0121] Voss, M. , Bange, H.W. , Dippner, J.W. , Middelburg, J.J. , Montoya, J.P. , and Ward, B. (2013) The marine nitrogen cycle: recent discoveries, uncertainties and the potential relevance of climate change. Philos Trans R Soc Lond B Biol Sci 368: 20130121–20130121. 23713119 10.1098/rstb.2013.0121 PMC3682741

[emi13682-bib-0122] Walsby, A.E. (1985) The permeability of heterocysts to the gases nitrogen and oxygen. Proc R Soc Lond Ser B‐Biol Sci 226: 345–366.

[emi13682-bib-0123] Ward, B.B. (2013a) Nitrification In Reference Module in Earth Systems and Environmental Sciences. Elsevier. Update of Encyclopedia of Ecology (2008), pp 2511–2518.

[emi13682-bib-0124] Ward, B.B. (2013b) How nitrogen is lost. Science 341: 352–353. 23888027 10.1126/science.1240314

[emi13682-bib-0125] Ward, B.B. , and Jensen, M.M. (2014) The microbial nitrogen cycle. Front Microbiol 5: 553. 25386170 10.3389/fmicb.2014.00553 PMC4208395

[emi13682-bib-0126] Watson, S.W. , Bock, E. , Valois, F.W. , Waterbury, J.B. , and Schlosser, U. (1986) *Nitrospira marina* gen. nov. sp. nov.: a chemolithotrophic nitrite‐oxidizing bacterium. Arch Microbiol 144: 1–7.

[emi13682-bib-0127] Welander, P.V. , Coleman, M.L. , Sessions, A.L. , Summons, R.E. , and Newman, D.K. (2010) Identification of a methylase required for 2‐methylhopanoid production and implications for the interpretation of sedimentary hopanes. Proc Natl Acad Sci U S A 107: 8537–8542. 20421508 10.1073/pnas.0912949107 PMC2889317

[emi13682-bib-0128] Wright, J.J. , Konwar, K.M. , and Hallam, S.J. (2012) Microbial ecology of expanding oxygen minimum zones. Nat Rev Microbiol 10: 1–14. 10.1038/nrmicro2778 22580367

[emi13682-bib-0129] Wuchter, C. , Abbas, B. , Coolen, M.J.L. , Herfort, L. , van Bleijswijk, J. , Timmers, P. , *et al* (2006) Archaeal nitrification in the ocean. Proc Natl Acad Sci 103: 12317–12322. 16894176 10.1073/pnas.0600756103 PMC1533803

[emi13682-bib-0130] Zehr, J.P. , and Kudela, R.M. (2011) Nitrogen cycle of the open ocean: from genes to ecosystems. Annu Rev Mar Sci 3: 197–225. 10.1146/annurev-marine-120709-142819 21329204

[emi13682-bib-0131] Zumft, W.G. (1997) Cell biology and molecular basis of denitrification. Microbiol Mol Biol Rev 61: 533–616. 9409151 10.1128/mmbr.61.4.533-616.1997 PMC232623

